# Supplementary material for: Electrostatic steering of thermal emission with active metasurface control of delocalized modes
Source: Nat Commun. 2024 Apr 20;15:3376. doi: 10.1038/s41467-024-47229-0 (PMC11032313; doi:10.1038/s41467-024-47229-0)
Supplement: Supplementary file 1 — Supplementary Information [file 41467_2024_47229_MOESM1_ESM.pdf]

Supplementary Information for

Electrostatic Steering of Thermal Emission with Active Metasurface Control of Delocalized Modes

Joel Siegel, Shinho Kim, Margaret Fortman, Changhao Wan, Mikhail A. Kats, Phillip W. C. Hon
Luke Sweatlock, Min Seok Jang,Victor Watson Brar

Correspondonce to: jang.minseok@kaist.ac.kr, vbrar@wisc.edu

This PDF file includes:

Supplementary Notes 1 to 7

1. Surface admittance of a metasurface
2. Analysis of plasmonic structure in the metasurface
3. Fabry-Perot model analysis
4. Higher order Fabry-Perot resonance peak
5. Analysis of potential factors affecting device performance
6. Potential angle steering capability
7. Modulation of angle-frequency spectra

Supplementary Note 1. Derivation of surface admittance of a metasurface

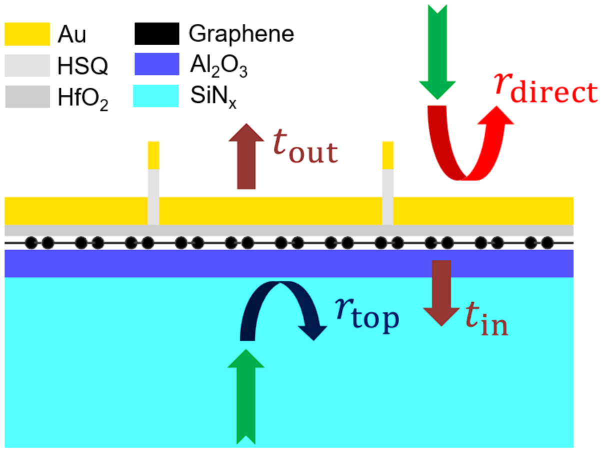

Fig. S1. Detailed configuration of metasurface and reflection/transmission coefficients.

The metasurface can be modeled as a thin conductive layer with effective surface admittance Y_s because the thickness of the metasurface is much thinner than the wavelength of the incident light[1, 2]. For normally incident light, Y_s can be solved for from the boundary condition $H_\mathrm{z}(0^+) - H_\mathrm{z}(0^-) = Y_\mathrm{s}E_\mathrm{x}(0)$. For the metasurface on top of the semi-infinite dielectric layer (Fig. S1), the normalized surface admittance $\tilde{Y}_\mathrm{s}$ is expressed in terms of transmission and reflection coefficients (t and r) of a metasurface as:

$$\tilde{Y}_\mathrm{s,t} = \frac{Y_\mathrm{s,t}}{Y_0} = \frac{Y_\mathrm{i}}{Y_0}(\frac{2}{t} - 1 - \frac{Y_\mathrm{t}}{Y_\mathrm{i}}) \tag{S1}$$

$$\tilde{Y}_\mathrm{s,r} = \frac{Y_\mathrm{s,r}}{Y_0} = \frac{Y_\mathrm{i}}{Y_0}(\frac{1-r}{1+r} - -\frac{Y_\mathrm{t}}{Y_\mathrm{i}}) \tag{S2}$$

Here, Y_0, Y_i and Y_t are the admittance of free space, input, and output media. For the top (bottom) excitation, Y_i and Y_t are air (SiN_x) and SiN_x (air), respectively. Figure S2(b) shows that the values of calculated admittance $\tilde{Y}_\mathrm{s,t}$ and $\tilde{Y}_\mathrm{s,r}$ are different. The surface admittance for both directions would be identical if the metasurface had zero thickness due to the same tangential electric fields at the top and bottom surfaces. However, the finite thickness of metasurface invalidates that condition and leads to two different surface admittance. In Note 1, we use normalized surface admittance derived from transmission coefficients.

We note that the dynamic behavior of the metasurface due to the Fermi level of graphene could be understood from the analysis of the surface admittance components. The real part (normalized surface conductance $\tilde{G}_\mathrm{s}$) and imaginary part (normalized surface susceptance $\tilde{B}_\mathrm{s}$) of the normalized surface admittance ($\tilde{Y}_\mathrm{s} = \tilde{G}_\mathrm{s} - i\tilde{B}_s$) provide information on absorption and scattering of the metasurface[3]. The normalized surface conductance $\tilde{G}_\mathrm{s}$ indicates the strength of the scattering process with the absorption of the metasurface. Because metal has much higher conductivity than graphene, the conductance of the metasurface is approximated to the conductance of graphene. Figure S2(c) shows higher conductance of the metasurface is obtained with an increase (decrease) of the Fermi level (frequency). In contrast, the

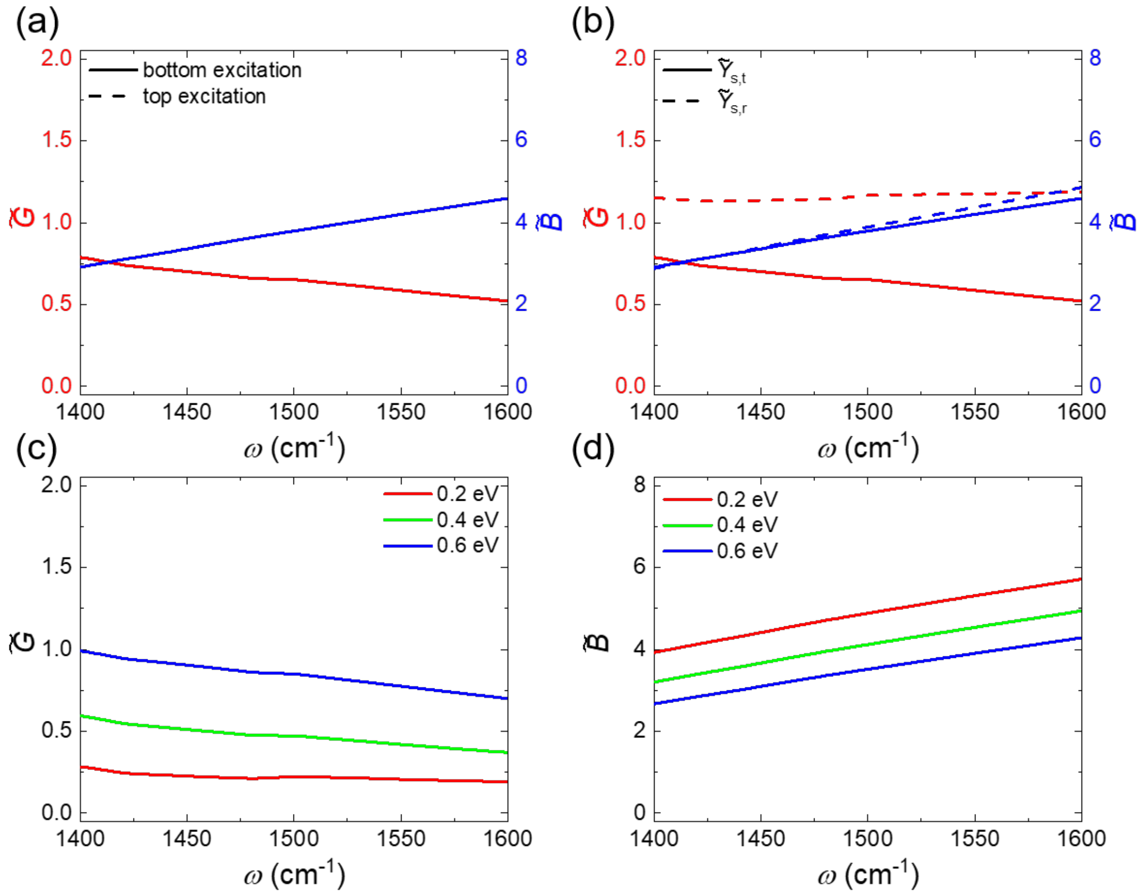

Fig. S2. Analysis of metasurface admittance. Conductance $\tilde{G}$ (left axis) and susceptance $\tilde{B}$ (right axis) of metasurface for different (a) excitation directions and (b) derivation variables (r and t) for E_F = $0.5\,\mathrm{eV}$. The (c) conductance $\tilde{G}$ and (d) susceptance $\tilde{B}$ for different Fermi levels of graphene.

normalized surface susceptance $\tilde{B}_\mathrm{s}$ indicates the strength of the scattering process without absorption of the metasurface. The susceptance of the metasurface is determined by a capacitance (C) and an inductance (L) derived from geometry and material parameters, where the surface susceptance B_s of metasurface is proportional to $(-i\omega L + (-i\omega C)^{-1})^{-1}$ with the convention of $e^{-i\omega t}$. Since the period of the metal slit array is much shorter than a free space wavelength, the surface susceptance of the metasurface shows a capacitive response. We note that the kinetic inductance of graphene is inversely proportional to the optical conductivity of graphene[4]. Thus higher Fermi level of graphene results in lowering surface susceptance of metasurface.

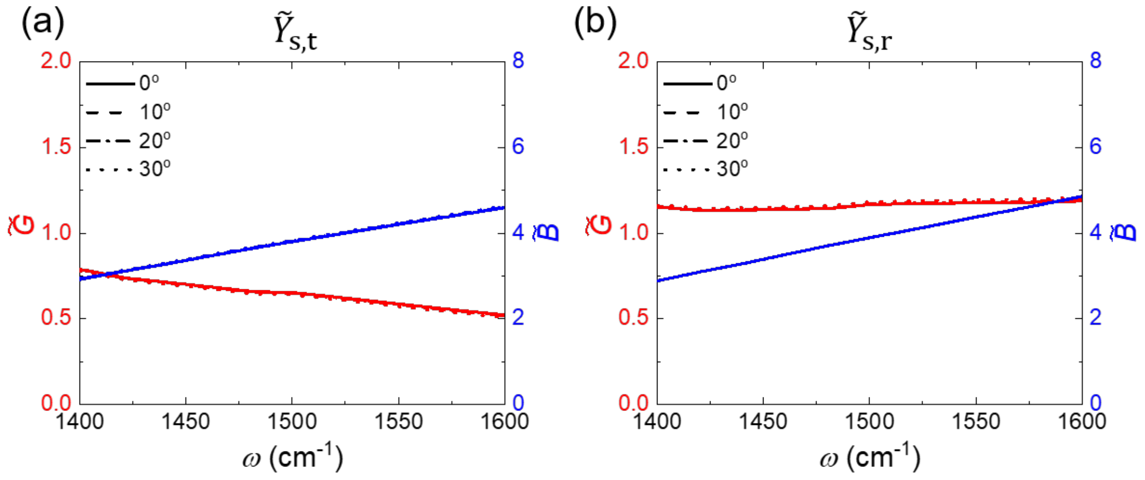

Fig. S3. Dependence of metasurface admittance on illumination directions. Conductance $\tilde{G}$ (left axis) and susceptance $\tilde{B}$ (right axis) of metasurface for different excitation directions derived from (a) transmission and (b) reflection coefficient.

Next, we calculate angle-dependent normalized surface admittance of the surface current which could be expressed in terms of angle-dependent transmission and reflection coefficients as:

$$\frac{Y_{\mathrm{s,t}}}{Y_{\mathrm{i}}} = \sec\theta_{\mathrm{t}}\left(\frac{2}{t} - \frac{\cos\theta_{\mathrm{t}}}{\cos\theta_{\mathrm{i}}} - \frac{Y_{\mathrm{t}}}{Y_{\mathrm{i}}}\right) \tag{S3}$$

$$\frac{Y_{\mathrm{s,r}}}{Y_{\mathrm{i}}} = \sec\theta_{\mathrm{t}}\left(\frac{\cos\theta_{\mathrm{t}}}{\cos\theta_{\mathrm{i}}}\frac{1-r}{1+r} - -\frac{Y_{\mathrm{t}}}{Y_{\mathrm{i}}}\right) \tag{S4}$$

where the angles θ_{i} and θ_{t} represent the angles of incidence and transmission, respectively. Figure S3 shows that the surface admittance remains constant regardless of the incident angle which is consistent with the ideal surface current model. This implies that the surface admittance derived from normal incident light could be exploited to derive angle-dependent reflection and transmission coefficients of the metasurface.

Supplementary Note 2. Analysis of plasmonic structure in metasurface

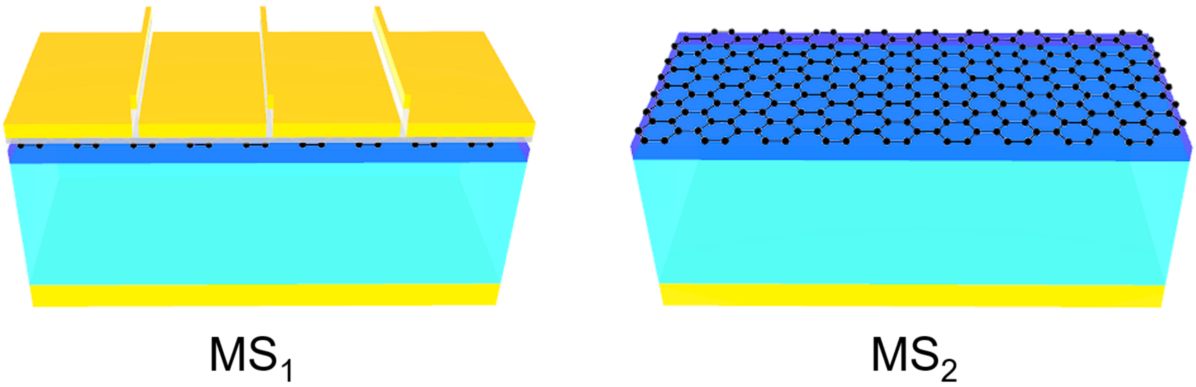

Fig. S4. Schematic of two different types of metasurfaces, MS_1 and MS_2, which differ based on the presence of a plasmonic metal slit array.

To investigate the impact of a plasmonic structure in the proposed graphene-metal hybrid metasurface, which serves as an electrically tunable mirror layer, we calculate the reflection coefficient for different geometric parameters of the plasmonic structure. The influence of the plasmonic structure is studied by comparing two different metasurface configurations MS_1 and MS_2 as shown in Fig. S4. Figure S5(a) and (b) illustrate the calculated amplitude ($|r_{\text{top, 0.3 eV}}|$) and phase difference ($\Delta\phi_{\text{top}} = \phi_{\text{top,0.3 eV}} - \phi_{\text{top,0 eV}}$) of reflection coefficients as a function of gap width g with Fermi levels $0\,\text{eV}$ and $0.3\,\text{eV}$. By comparing Fig. S5(a) and (c), we observe that the amplitude of the reflection coefficient is mainly influenced by the slit width w, which represents the surface coverage of the highly reflective metal film. On the other hand, the phase modulation for the Fermi level is determined by the electric field intensity at the surface of graphene, which is determined by the compressed transmitted electromagnetic wave as the form of plasmonic wave. As a result, the phase difference is significantly affected by both geometric parameters. We emphasize that the shape of the resonance peak in the Fabry-Perot (F-P) resonator and the resonance frequency shift for the Fermi level modulation are determined by the reflection coefficient of the metasurface. Therefore, proper metasurface design is crucial for achieving a sufficiently high and sharp emissivity peak with a considerable resonance frequency shift for Fermi level modulation.

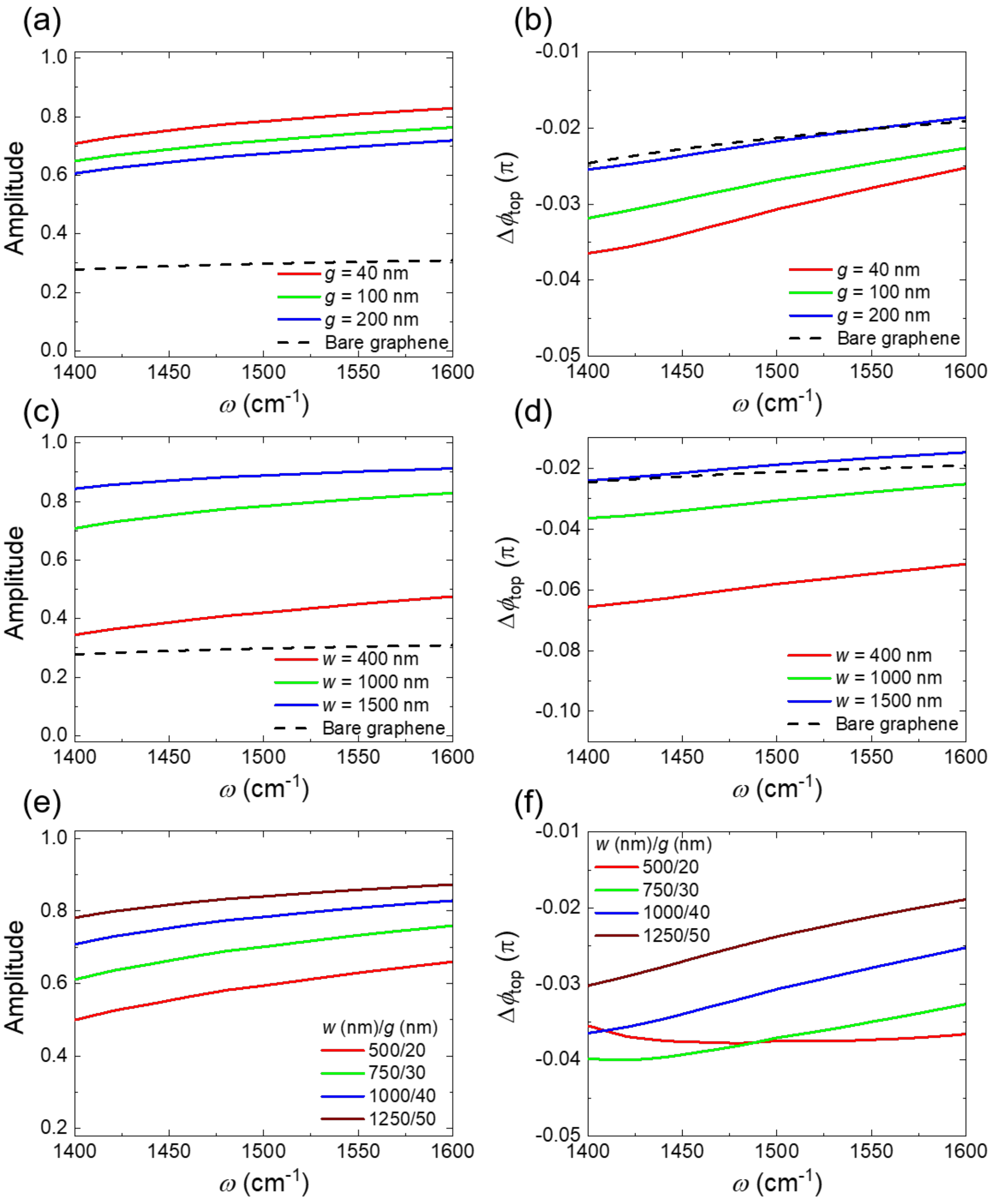

Fig. S5. Influence of plasmonic metasurfaces on reflection coefficient modulation. The amplitude $|r_{\text{top},0.3\,\text{eV}}|$ and phase difference $\Delta\phi_{\text{top}} = \phi_{0.3\text{ eV}} - \phi_{0\text{ eV}}$ for slit (a, b) and gap (c, d) widths g and w, respectively. The black dashed line shows the reflection coefficient of bare graphene structure MS_2. The amplitude (e) and phase difference (f) of metasurface MS_1 for constant ratio $(g/w) = 25$.

Supplementary Note 3. Fabry-Perot model analysis

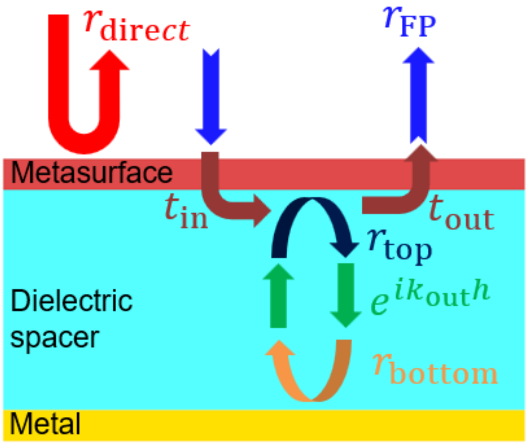

Fig. S6. Schematic of F-P model for a structure consisting of graphene-plasmonic metasurface/thick dielectric spacer/metal reflector.

In order to deeply understand the operation mechanism of the proposed structure, we develop the semi-analytical solution of the reflection coefficient for the proposed structure based on F-P interference (Fig. S6). To simplify the reflection coefficient equation of the structure, the dielectric film stack is merged into a single film, where reflection at the bottom interfaces of Al_2O_3 and HfO_2 films are ignored. The graphene layer thickness is excluded from the thickness of the dielectric stack because it was modeled as a zero-thickness conductivity sheet layer. Note that the subwavelength period and the non-resonant response of the metasurface suppress higher-order diffraction and deflection for incident light. We directly calculate the reflection (r) and transmission coefficients (t) from the definition in electromagnetic wave theory. The F-P reflection coefficient of the proposed structure is

$$r_{\text{FP}} = \frac{t_{\text{in}} t_{\text{out}} r_{\text{bottom}} e^{2ik_{\text{out}}h}}{1 - r_{\text{top}} r_{\text{bottom}} e^{2ik_{\text{out}}h}} \tag{S5}$$

where k_{out} and h are the out-of-plane wavevector and the thickness of the dielectric layer. The wavevector k_{out} is a function of refractive index and incident angle, and thus total phase accumulation is sum of $k_{\text{out}}h$ at each film.

We note that a rich free electron density of noble metal makes it difficult to obtain a noticeable electro-optic effect in the bottom and top metal layers. Therefore, the modulation of the resonant frequency of the proposed structure is determined by the Fermi-level dependent surface admittance of the metasurface. Figure S7 shows the reflection and transmission coefficients for normally incident TM polarized plane wave as a function of Fermi levels. In the extreme case ($E_F \to 0\,\text{eV}$), the amplitude and phase of r_{top} has high value of 0.82 and 0.83π which is close to planar metal film because metal slit array covers $> 95\%$ of surface area. In contrast, the increase (decrease) of conductance (susceptance)

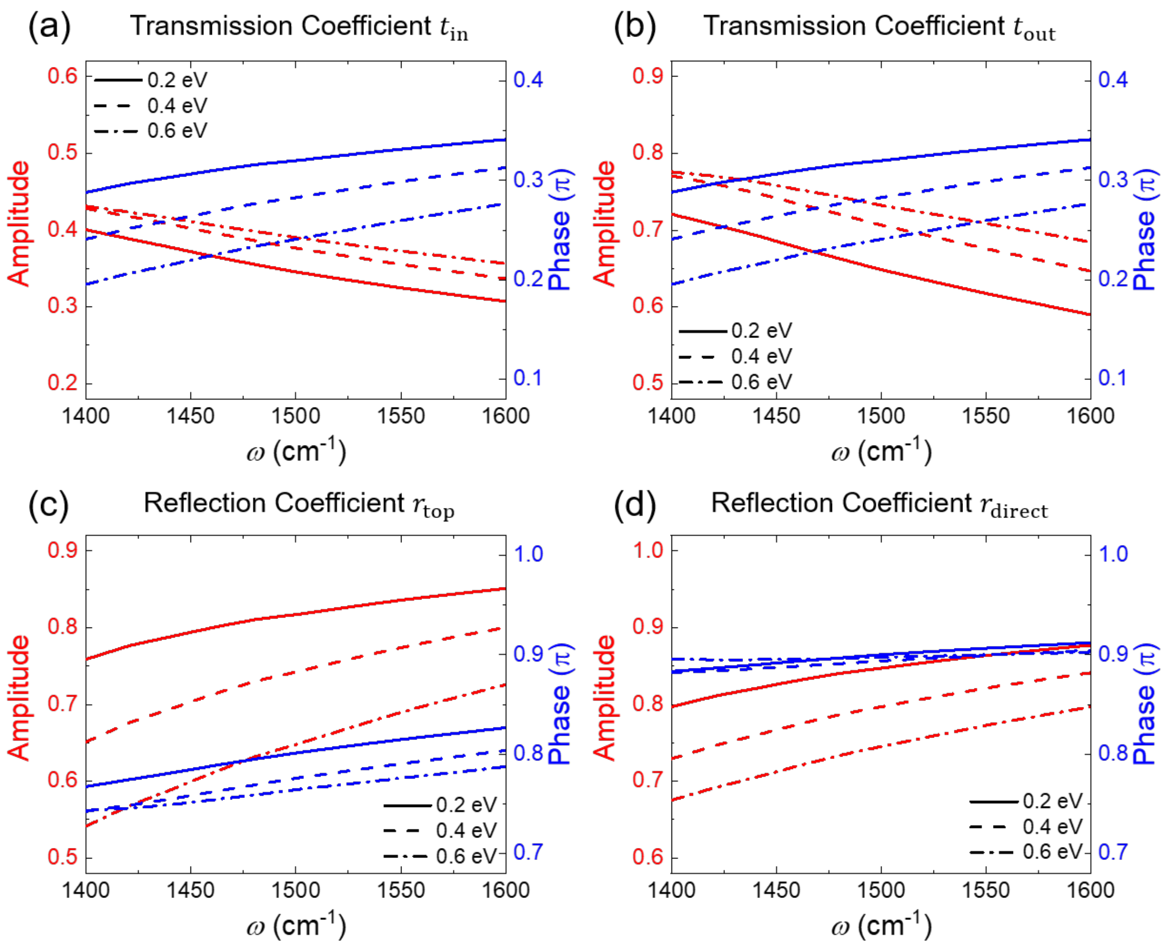

Fig. S7. Fermi level dependence of transmission/reflection coefficients. The amplitude (left axis) and phase (right axis) of transmission (a,b) and reflection (c,d) coefficients for different Fermi levels.

of the metasurface reduces the amplitude and phase of reflection coefficients for higher E_F or lower ω. Similarly, the transmission coefficients $t_{\text{in,out}}$ also transit from a metal mirror-like response to a lossy dielectric response for E_F and ω.

In the F-P model, the total absorption A can be calculated as $1 - |r_{\text{tot}}|^2 = 1 - |r_{\text{direct}} + r_{\text{FP}}|^2$. Note that the amplitude and phase variation ($r_{\text{direct}}/\phi_{\text{direct}}$) of the direct reflection is significantly smaller than the amplitude and phase ($r_{\text{FP}}/\phi_{\text{FP}}$) of the Fabry-Perot reflection as shown in Fig. S8(a) and (b). Thus, the dynamic behavior of total absorption is primarily determined by $r_{\text{FP}}/\phi_{\text{FP}}$. The dynamic behavior of these coefficients can be understood by analyzing the dependence of F-P reflection spectra r_{FP} on the Fermi level and the incident angle. The resonance frequency of r_{FP} is determined by the phase condition $2k_{\text{out}}h + \phi_{\text{top}} + \phi_{\text{bottom}} = 2\pi m$, where ϕ_{top} and ϕ_{bottom} are the phase of reflection coefficient r_{top} and r_{bottom} respectively, and m is an integer. Given the nearly constant reflection phase of the bottom electrode, the reflection phase change of metasurface $\Delta\phi_{\text{top}}$ is compensated by the change of out-of-plane wavevector k_{out}, which contributes to propagation phase $\phi_{\text{p}} = 2\Delta k_{\text{out}}h$. As ϕ_{top} is inversely proportional to the Fermi level, the k_{out} at resonance frequency should be increased for higher Fermi level, leading to a blue shift in F-P resonance. Figure S8(d) shows that the resonance frequency ω_r

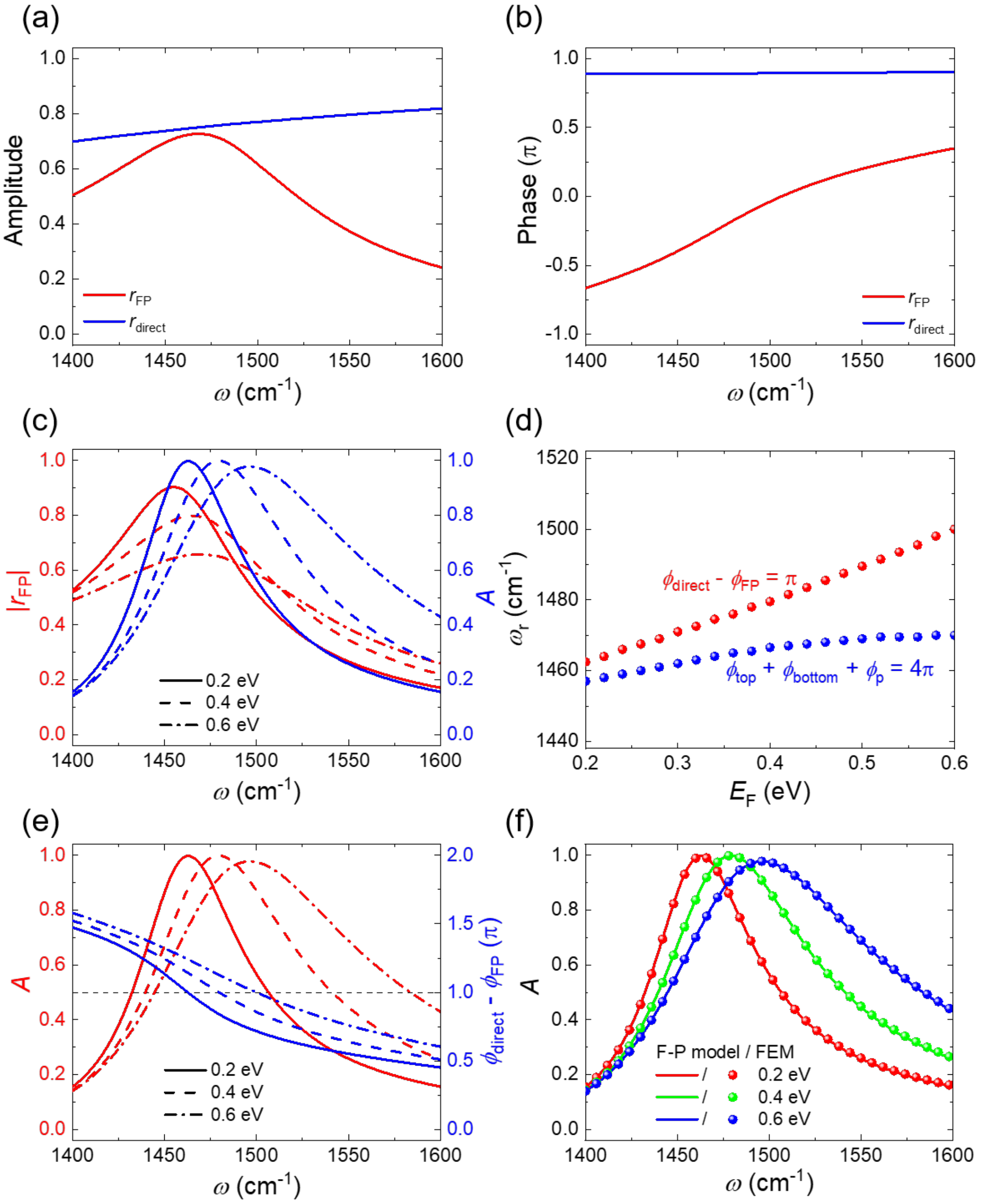

Fig. S8. Analysis of Fermi level dependence using the semi-analytical model. The (a) amplitude and (b) phase of reflection coefficient r_{FP} and r_{direct} for Fermi level E_F = 0.5 eV (c) The amplitude of r_{FP} (left axis) and total absorption derived from F-P model (right axis). (d) The resonance frequencies ω_r and ω_{FP} as a function of graphene Fermi levels. (e) The F-P model total absorption (left axis) and phase difference (right axis) for different Fermi levels of graphene. (f) The total absorption from FEM-based full wave simulation and F-P model.

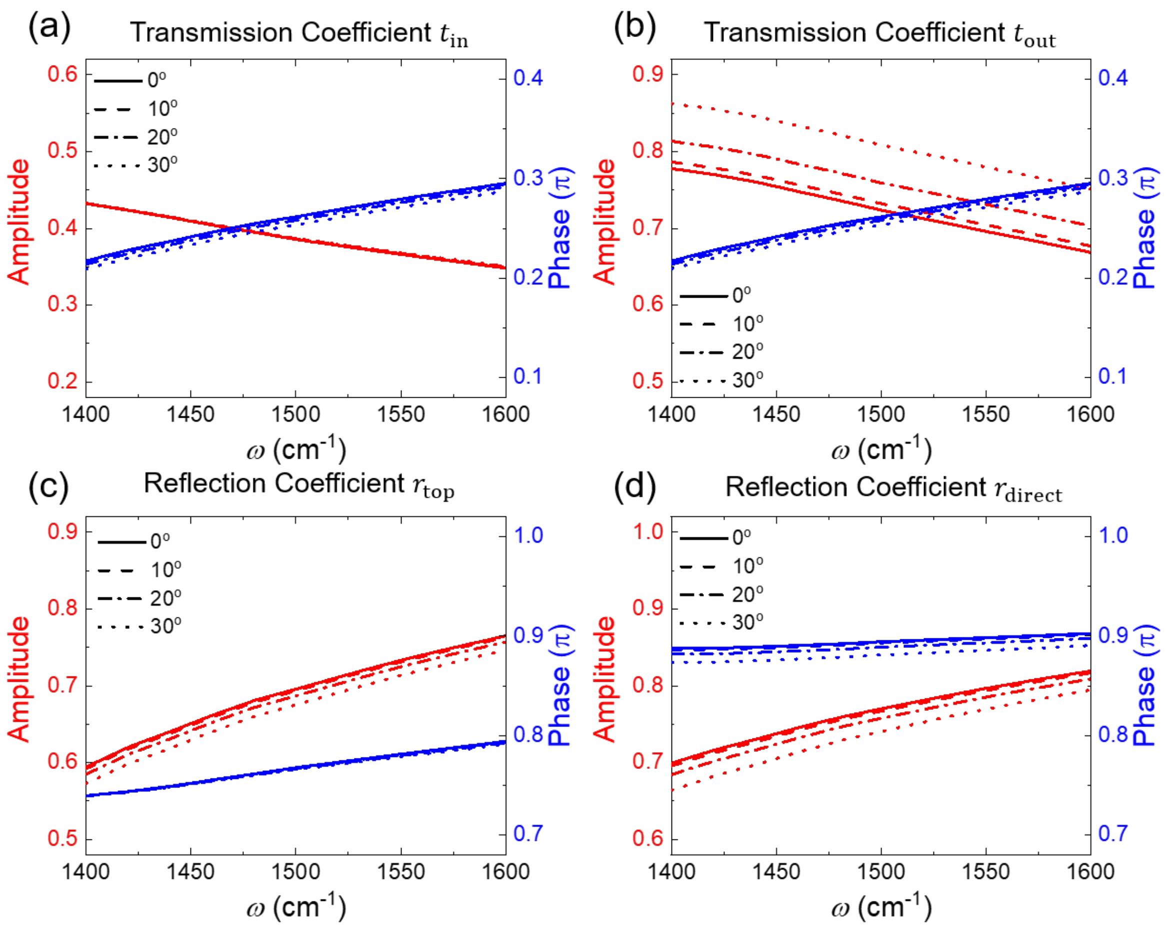

Fig. S9. Incident angle dependence of transmission/reflection coefficients. The amplitude (left axis) and phase (right axis) of transmission (a,b) and reflection (c,d) coefficients for different incident angles.

derived from the phase condition for maximum absorption ($\phi_{\text{direct}} - \phi_{\text{FP}} = \pi$) is larger than the F-P resonance frequency ω_{FP}, and that the change of ω_r is faster than ω_{FP} for Fermi level modulation. At the F-P resonance condition, the phase of r_{FP} is equal to the sum of phases of transmission coefficients t_{in} and t_{out} where the π phase difference condition is not satisfied. Since $\phi_{\text{direct}} - \phi_{\text{FP}} > \pi$ and $\partial(\phi_{\text{direct}} - \phi_{\text{FP}})/\partial\omega < 0$, the resonance frequency of maximum absorption ω_r becomes greater than F-P resonance frequency ω_r. In addition, phase modulation of r_{direct}, t_{in}, and t_{out} for Fermi level provides additional phase difference between ϕ_{direct} and ϕ_{FP}, thus the resonance frequency shift of $\Delta\omega_r$ is larger than $\Delta\omega_{\text{FP}}$.

On the other hand, the parameters and variables in the F-P reflection formula also depend on the incident angle θ of the excitation light. Figure S9 shows the incident angle-dependent reflection and transmission coefficients. In the discussion of angle-dependent calculation, the incident angle is defined as the angle in the air. The amplitude and phase of coefficients show slower variation for incident angles than the Fermi level of graphene. Larger amplitude change of t_{out} than t_{in} originates from the input admittance difference (Eq. S1 and 2). We emphasize that the shift of F-P resonance frequency comes from phase accumulation change of propagating wave ($2\Delta k_{\text{out}}h$) because the change of reflection and transmission coefficients for incident angles are smaller than the change of out-of-plane wavevector. The

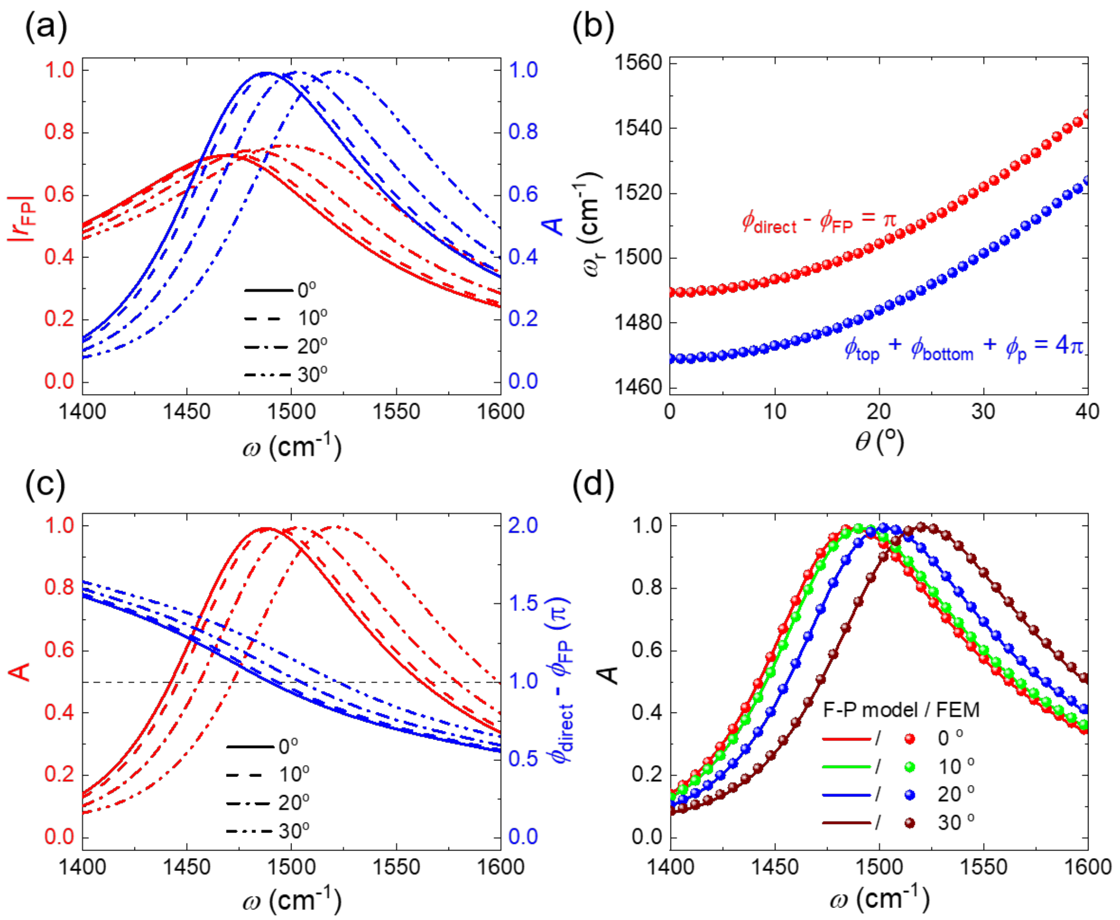

Fig. S10. Analysis of incident angle dependence using the semi-analytical model. (a) The amplitude of r_{FP} (left axis) and total absorption derived from F-P model (right axis) for different incident angles. (b) The resonance frequencies ω_r and ω_{FP} as a function of incident angles. (c) The F-P model total absorption (left axis) and phase difference (right axis) for different Fermi levels of graphene. (d) The total absorption from FEM-based full wave simulation and F-P model.

out-of-plane wavevector is given as $k_{\mathrm{d}}\cos\theta_{\mathrm{d}} = k_{\mathrm{out}}$ where k_{d} and θ_{d} are the wavevector and the propagation angle in the dielectric spacer. Since the propagation angle has the relation with incident angle θ as $n_{\mathrm{d}}\sin\theta_{\mathrm{d}} = \sin\theta$, a larger incident angle reduces propagation phase accumulation. To compensate for the phase decrease by $\cos\theta_{\mathrm{d}}$, k_{d} should be increased, which is equivalent to a blue shift of resonance frequency. Unlike the resonance frequency change for Fermi level modulation, the resonance frequency change of $\Delta\omega_r$ and $\Delta\omega_{\mathrm{FP}}$ for incident angle are similar due to the difference in the phase modulation method as shown in Fig. S10(a).

We note that the maximum absorption phase condition, $\phi_{\mathrm{direct}} - \phi_{\mathrm{FP}} = \pi$, is worked when the amplitude of resonant mode is comparable with non-resonant direct reflection at the resonance frequency. The strength of F-P resonance is inversely proportional to the Fermi level due to increased free carrier absorption in the graphene (Fig. S11(a)). Therefore, this assumption cannot be satisfied at a high Fermi level, and the difference between resonance frequencies, ω_r, derived from the phase condition and the model-based calculation becomes larger as the increase of Fermi level (Fig. S11(b)). On the other

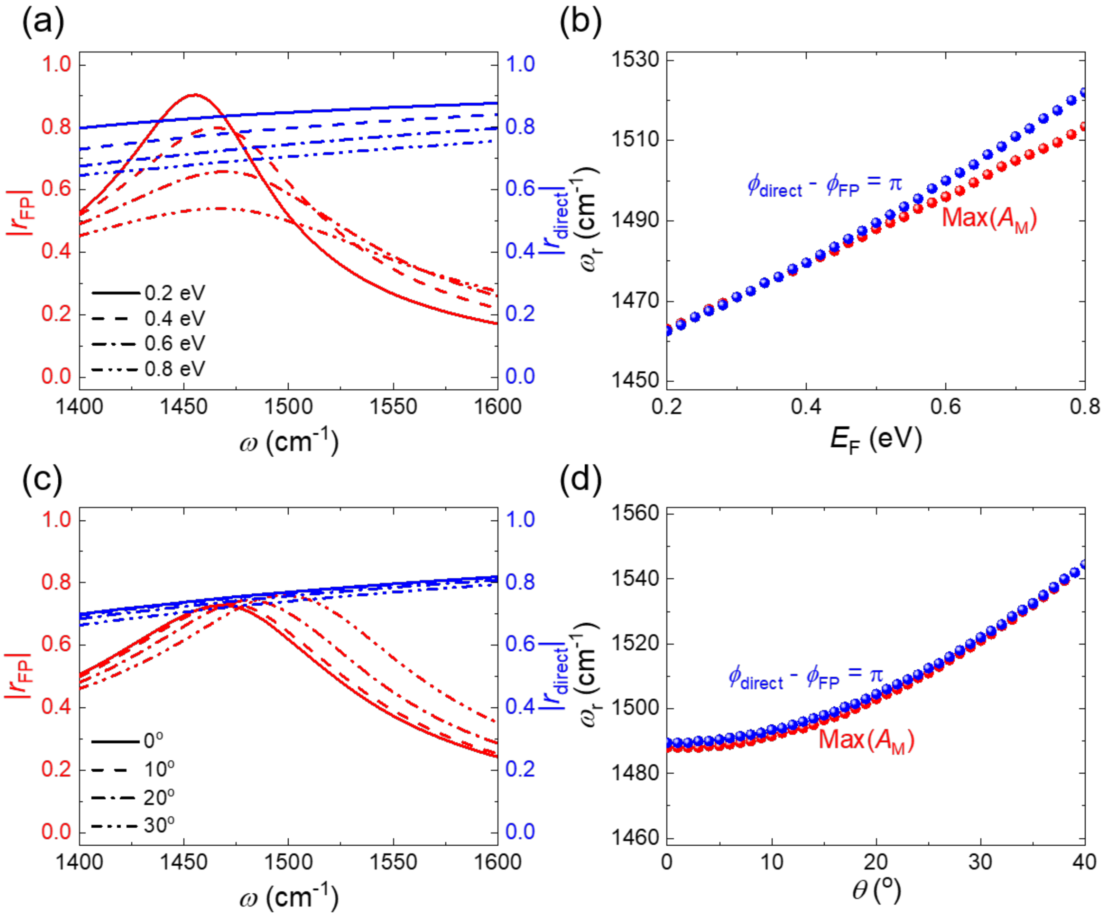

Fig. S11. Range of applicability for the semi-analytical model: Fermi level and incident angle. The amplitude of r_{FP} and r_{direct} for different (a) Fermi levels of graphene and (c) incident angles. The resonance frequencies ω_r and ω_{FP} as a function of (b) Fermi levels and (d) incident angles.

hand, the maximum absorption phase condition is worked regardless of the incident angle due to the conservation of F-P resonance strength (Fig. S11(c)).

Calculation of emission angle at measured frequency is required to obtain desired functionality of the proposed device. However, this process requires the calculation of angular spectra for broad frequency spectrum and various Fermi levels. Instead of finite element method(FEM)-based full-wave angular spectrum calculation, we try to obtain angular spectrum from reflection and transmission coefficients derived from the surface admittance model. The Fresnel coefficients of the graphene metasurface are described by the following equations:

$$r = \frac{\tilde{Y}_{\text{i}}\cos\theta_{\text{t}} - \tilde{Y}_{\text{t}}\cos\theta_{\text{i}} - \tilde{Y}_{\text{s,r}}\cos\theta_{\text{i}}\cos\theta_{\text{t}}}{\tilde{Y}_{\text{t}}\cos\theta_{\text{i}} + \tilde{Y}_{\text{i}}\cos\theta_{\text{t}} + \tilde{Y}_{\text{s,r}}\cos\theta_{\text{i}}\cos\theta_{\text{t}}} \tag{S6}$$

$$t = \frac{2\tilde{Y}_{\text{i}}\cos\theta_{\text{i}}}{\tilde{Y}_{\text{t}}\cos\theta_{\text{i}} + \tilde{Y}_{\text{i}}\cos\theta_{\text{t}} + \tilde{Y}_{\text{s,t}}\cos\theta_{\text{i}}\cos\theta_{\text{t}}} \tag{S7}$$

The calculated total absorption derived from these coefficients agrees well with the absorption calculated using FEM, as depicted in Fig. S12(a).

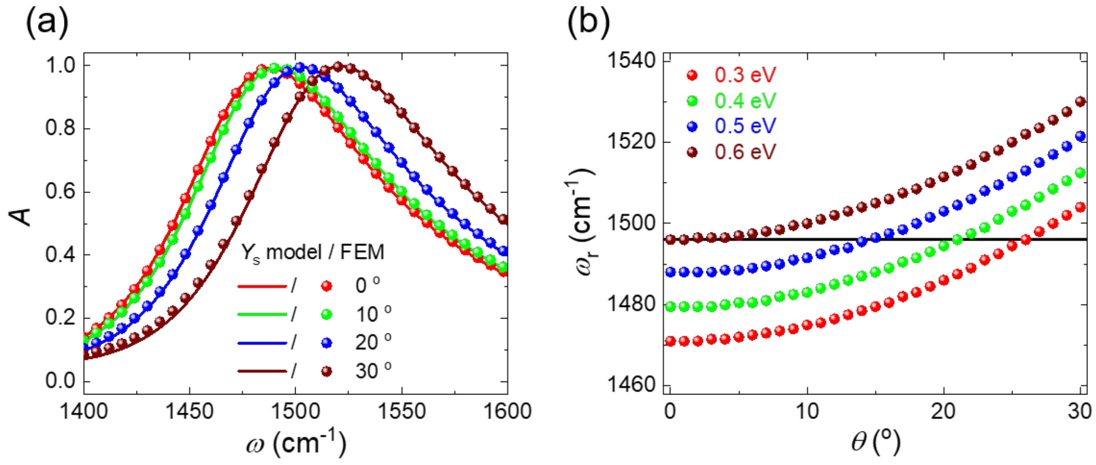

Fig. S12. Validation of the surface admittance-based F-P model. (a) The total absorption from FEM-based full wave simulation and surface admittance-based F-P model. (b) The resonance frequency ω_r as a function of incident angles for various Fermi levels of graphene. The black line indicates the resonance frequency for $E_F = 0.6\,\mathrm{eV}$.

As a further step, we developed a graphical method that enables the rapid identification of required Fermi levels to achieve the desired emission angle at a given operating frequency. Figure S12(b) illustrates the calculated resonance frequencies ω_r of the device as a function of the incident angle of light. In this plot, we include a straight line with the measurement frequency as the y-intercept. The intersection point of the resonance frequency curve and the straight line indicates the emission angle at the measurement frequency. This approach allows for the rapid estimation of the emission angle, which is essential for thermal emission steerer design.

Supplementary Note 4. Higher order Fabry-Perot resonance peak

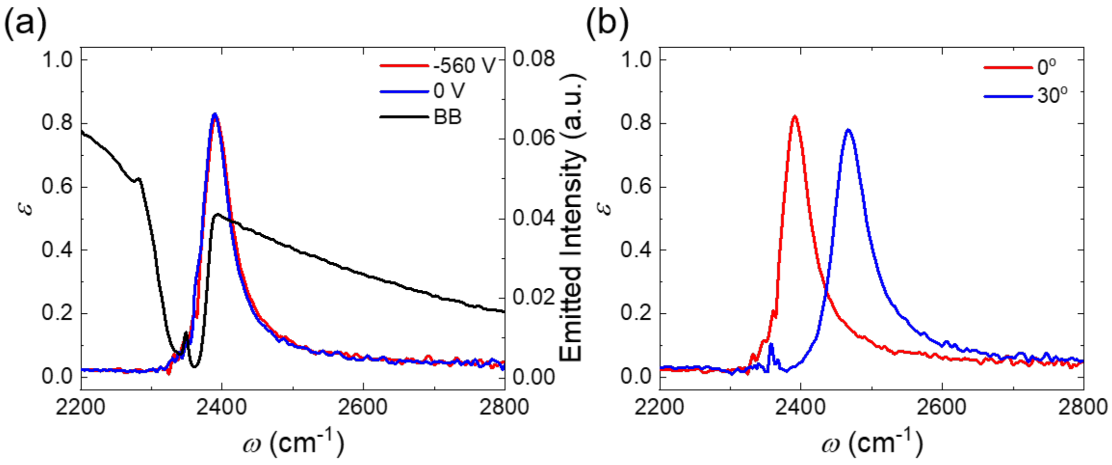

Fig. S13. Emission spectra in the high frequency regime The measured emission spectrum in high frequency regime as a function of (a) applied voltages and (b) incident angles.

Supplementary Note 5. Analysis of potential factors affecting device performance

A. Elemental absorption analysis

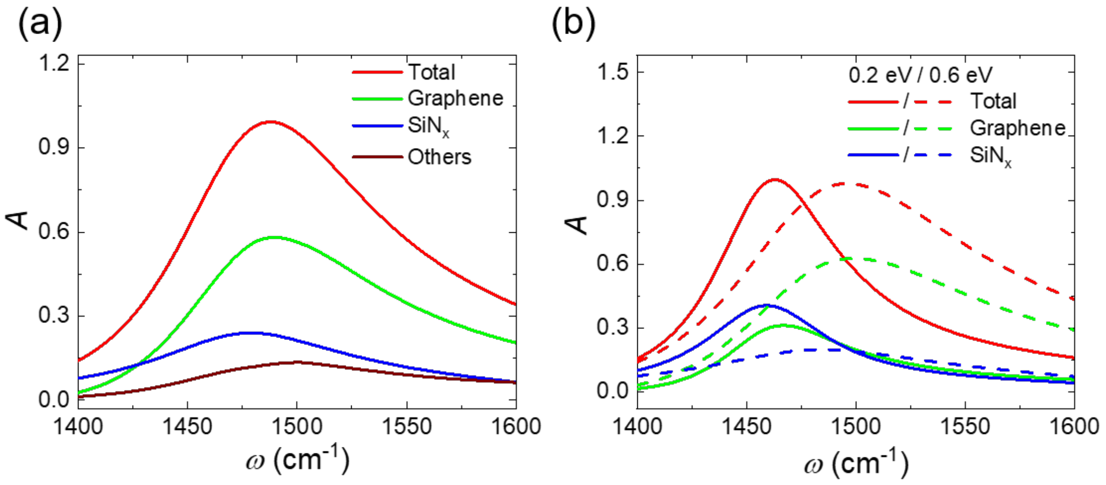

Fig. S14. Elemental absorption analysis of the device. (a) The absorbed power by different material elements with $E_F = 0.5\,\mathrm{eV}$. (b) The total, graphene and SiN_x absorption for $E_F = 0.2\,\mathrm{eV}$ and $E_F = 0.6\,\mathrm{eV}$.

To deeply understand the resonance behavior in the F-P resonator with a non-resonant metasurface, we performed FEM simulation to calculate elemental absorptions. The absorption of elements was calculated from $\frac{1}{P_0}\int_S \frac{1}{2}\mathrm{Re}(\mathbf{J}\cdot\mathbf{E}^*)dS$ for graphene sheet and $\frac{1}{P_0}\int_S \frac{\omega}{2}\mathrm{Im}(\epsilon_c)|\mathbf{E}|^2 dS$ for finite thickness films where P_0, $\mathbf{J}$, $\mathbf{E}$, and ϵ_c are incident wave power, current density, electric field, and complex permittivity of materials. Figure S14(a) shows the elemental absorption of Graphene, SiN, and other components. It is important to emphasize that the dominant absorption occurs in the graphene sheet and $\mathrm{SiN_x}$ membrane. Consequently, the total absorption peak is formed by the summation of these two absorption components. As a result, the resonance peak of the total absorption exhibits a broader frequency range and a larger shift than a single F-P resonance peak, as illustrated in Fig. S14(b).

B. Geometry and material parameters

We analyze the impact of material and geometry on the device caused by imperfect fabrication. Figure S15(a) shows the total absorption of the device for various geometric parameters. The deviation in structural parameters was determined by considering the fabrication tolerance specific to each fabrication process. Among the geometrical parameters, the gap width, slit width, and HfO_2 thickness exhibit noticeable resonance peak frequency shifts and broadening. This is because electromagnetic energy density at the surface of graphene is influenced by two geometric factors: exposed graphene area (gap width/slit width) and metal-graphene distance (HfO_2 thickness). Even a $2\,\mathrm{nm}$ thickness variation in HfO_2 thickness considerably alters the modulation performance of resonance frequency. The broadening of the resonance peak is directly proportional to the Fermi level due to enhanced free carrier absorption. In contrast, variation in the thickness of other elements (Al_2O_3, SiO_x, and Slit) have negligible effects on the optical properties of the device.

To investigate the impact of deviations in material optical properties, we performed calculations of the total absorption for different carrier mobilities (graphene) and permittivities (Al2O3, SiO_x, and HfO2). Figure S16(a) shows that at moderate carrier mobilities ($> 300\,\mathrm{cm}^2/\mathrm{V{\cdot}s}$), the resonance peak of the total absorption exhibited tolerance to deviations. However, excessively low carrier mobilities broaden the resonance peak and decrease the resonance frequency shift for Fermi level modulation. Considering the potential damage induced by fabrication processes such as dielectric deposition and e-beam exposure, the lower modulation performance observed in the fabricated devices of this project could be attributed to this effect. On the other hand, for calculating the total absorption spectra of the device for different frequency-dependent permittivity $K\epsilon_\mathrm{r}$, where K is the scaling factor, it was observed that there is a small resonance frequency shift with a slight change in broadness for high permittivity deviations. Therefore, in the proposed scheme, the deviation in material properties of dielectric layers has minimal effect on the calculation of the total absorption.

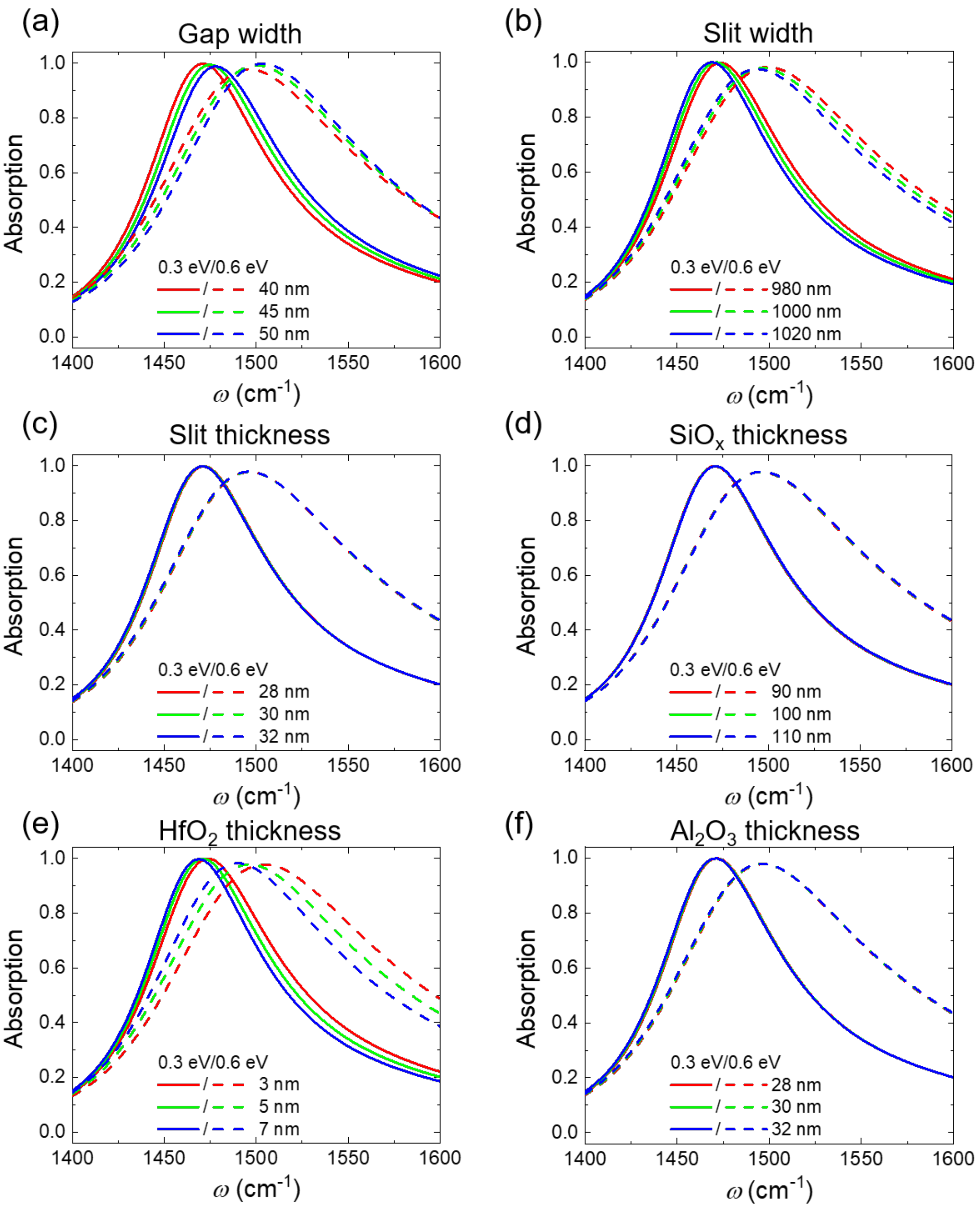

Fig. S15. Deviation analysis of structural parameters in the device. The total absorption for different geometric parameters (a) gap width, (b) slit width, (c) slit thickness, (d) SiO_x thickness, (e) HfO_2 thickness, and (f) Al_2O_3 thickness with E_F = 0.3 eV and 0.6 eV, respectively.

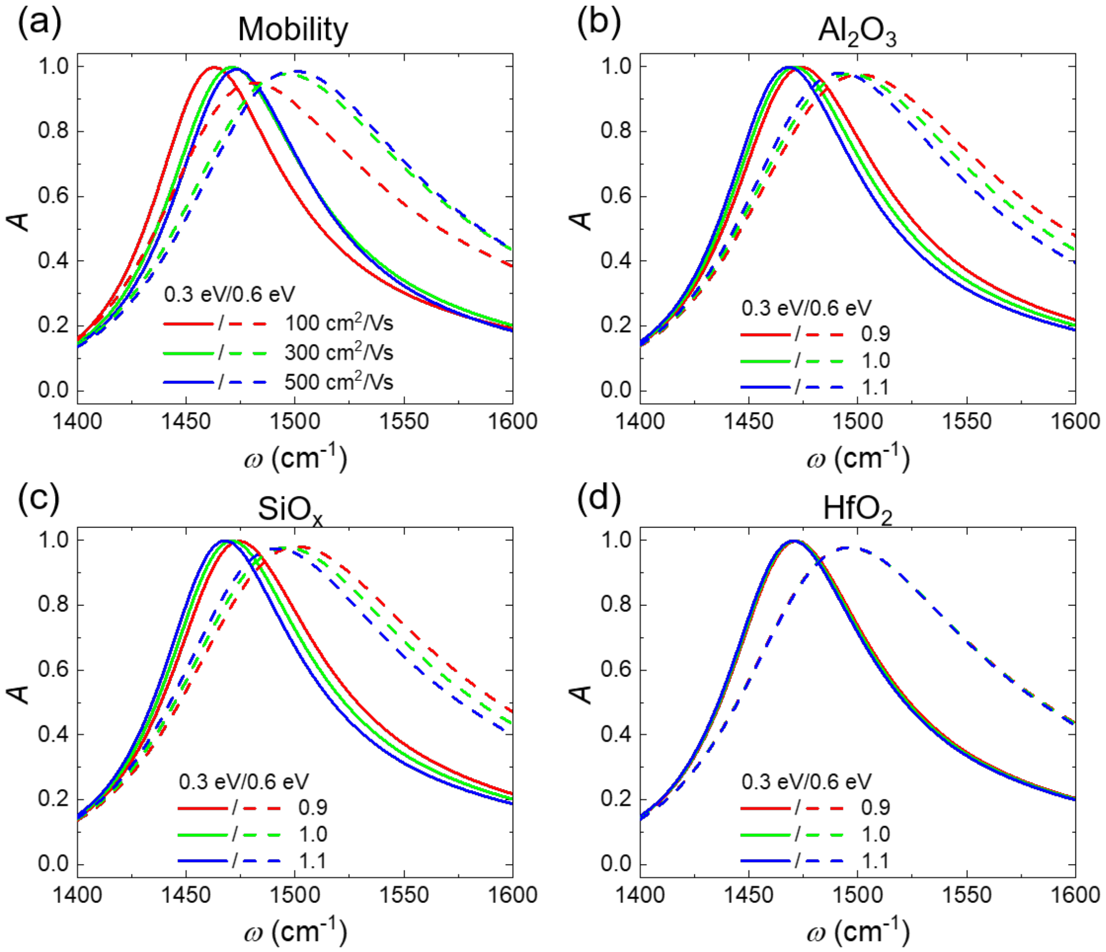

Fig. S16. Deviation analysis of optical properties of materials in the device. The total absorption for different material parameters (a) carrier mobility of graphene, the real part of permittivity of (b) Al_2O_3, (c) SiO_x, and (d) HfO_2 with $E_F = 0.3\,\mathrm{eV}$ and $0.6\,\mathrm{eV}$ for different scaling factor K, respectively.

C. Nonclassical effects in metal-graphene interaction

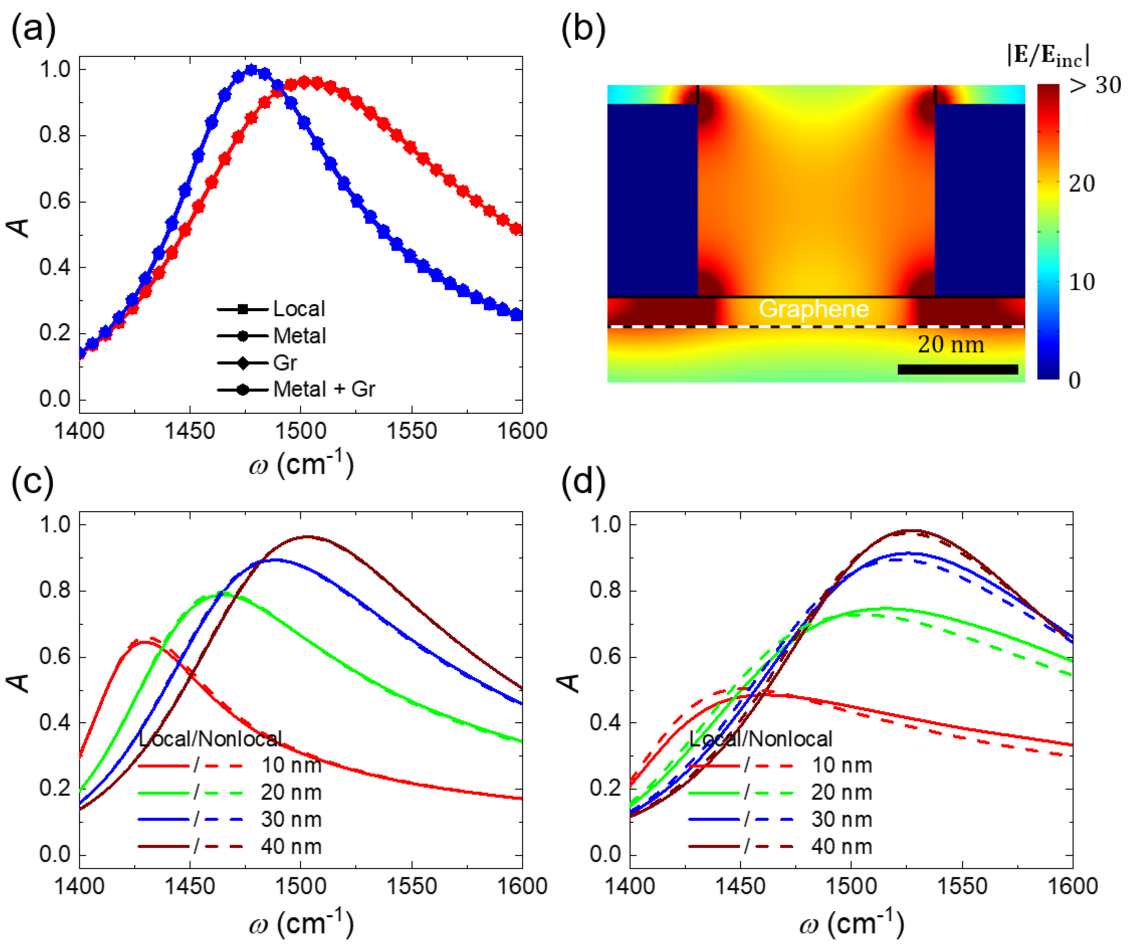

Fig. S17. Analysis of nonlocal effect in the device. (a) The total absorption for different models with $E_F = -0.38\,\text{eV}$ (blue) and $-0.68\,\text{eV}$ (red). (b) The normalized magnitude of electric field distribution at resonance frequency with $E_F = -0.68\,\text{eV}$. The white dot line is zero thickness graphene layer. The total absorption of local and nonlocal models for different gap sizes with (c) $5\,\text{nm}$ and (d) $1\,\text{nm}$ of HfO_2 thickness.

For the proposed structure, the length scale of metallic structures (graphene and slit array) is close to the electron wavelength in materials. Therefore, nonclassical effects that are not considered in classical electromagnetic simulations, such as the nonlocal effect, quantum tunneling effect, and quantum confinement effect, need to be taken into account. Additionally, the $30\,\text{nm}$ thickness of metal slit is thick enough to avoid optical property change by the quantum confinement effect[5]. In the case of the quantum tunneling effect between graphene-metal structures, it becomes observable for gap distance less than $1\,\text{nm}$ [6]. Thus, $5\,\text{nm}$ passivation HfO_2 layer between the metal slit array and graphene does not enter the quantum tunneling regime. Therefore, the nonclassical effect of concern is the nonloal effect, which has been observed in metallic structures on the scale of a few tens of nanometers. This effect arises from the nonideal spatial concentration of electrons due to quantum pressure in electron wave functions.

To investigate the nonlocal effect, we employ a hydrodynamic model for Au slit array and graphene sheet. The currents density **J** inside metal and graphene sheet induced by electric field **E** with frequency ω can be described by the following equation in the nonlocal frame[7, 8]:

$$\beta^2\nabla(\nabla\cdot\mathbf{J})+(\omega^2+i\gamma\omega)\mathbf{J})=i\omega\omega_p^2\epsilon_0\mathbf{E} \tag{S8}$$

where ϵ_0, γ and ω_p are the vacuum permittivity, damping coefficient and plasma frequency, respectively. The nonlocal parameter, β, depends on the Fermi level and dimensionality. We solve the equation using PDE and wave optics modules in COMSOL commericial FEM software. Figure S17(a) compares the total absorption of the device for different simulation configurations: full local, graphene nonlocal, metal nonlocal and full nonlocal. The results indicate that the fabricated device is rarely affected by the nonlocal effect. This can be attributed to two factors: (1) non-resonant scattering of the metal slit array (2) the increased effective gap width due to electric field spreading. However, when the thickness of HfO_2 is decreased to $1\,\mathrm{nm}$, the nonlocal effect starts to affect the resonance peak due to increased electric field confinement in the gap region.

D. Leakage current in dielectric spacer

In the classical capacitor model, the assumption in calculating induced charge density is that the applied voltage is the same value as the voltage drop along the dielectric films sandwiched by the two electrode pairs. If the voltage drops across other parts of the device are significant, the estimated induced charge density by the capacitor model may be higher than the actual charge density during the operation of the device. Thus, the inefficient gating of graphene by this factor diminishes the angle steering range and the associated spectral peak shift. To investigate the degradation of the device operation caused by inefficient gating, we measured the leakage current in the SiN_x/Al_2O_3 layer at $250\,^{\circ}C$, shown in Fig. S18. The measured resistance across the dielectric spacer ($> 10^2$ MΩ) is many orders of magnitude higher than any electrical contact in our circuit and the resistance of the graphene sheet ($100 \sim 300$ Ω). Consequently, the gate voltage drop occurs almost entirely across the SiN_x/Al_2O_3, despite the small leakage current.

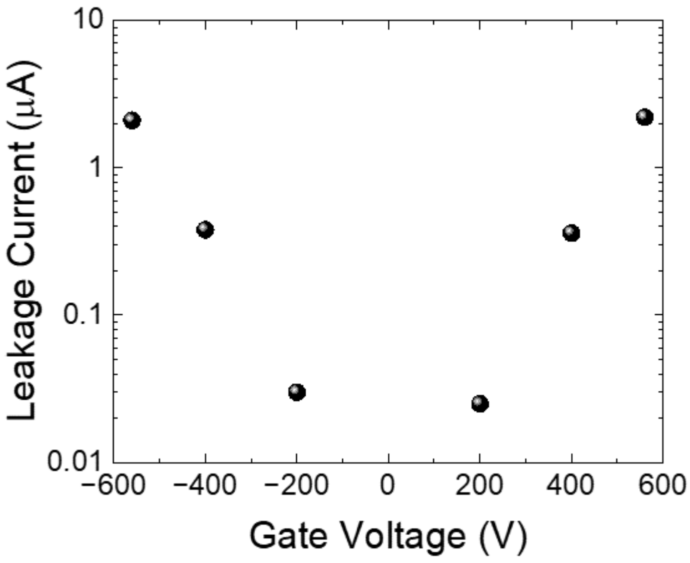

Fig. S18. Measurement of leakage current across the SiN_x/Al_2O_3 layers between the graphene sheet and the bottom Au backgate. The device temperature is maintained at $250\,^{\circ}C$ during measurement.

E. Dependence of gate capacitance on temperature and gate voltage

The capacitance between the graphene and the backgate is determined by the static dielectric constants of the dielectric materials. The electrostatic dielectric constant of the membrane layer could vary with temperature and gating voltage. Therefore, it is important to investigate the effects of the aforementioned factors to understand the discrepancy of emissivity spectra in measurement and calculation. For the SiN_x substrates used in this work, measurements in our previous study have shown that the dielectric constant increases only slightly with temperature up to $250\,^{\circ}$C, and remains unchanged by the applied gate voltage[9]. These measurements justify the basic capacitance model used in estimating how carrier density changes with applied gate voltage, which assumes the dielectric constants of the SiN_x/Al_2O_3 to be independent of temperature and gate voltage.

F. Uncertainty of dielectric constant

Commercially available products have a variance of dielectric constant owing to the fluctuation of growth conditions. In calculating induced carrier density at the surface of graphene by gating, the dielectric constant of the SiNx memebrane layer was assumed to be 7.5, as derived in our previous work[1]. However, the dielectric constant of the purchased product has a variance of 1, corresponding to $\sim$15% of dielectric constant. Considering this variance, the magnitude of the induced carrier density could be reduced to 20% of our expectation.

G. Impurity and charge trap effect

One of the important issues in the graphene-based active metasurface is charge traps and atmospheric impurities on or in the SiN_x/Al_2O_3 which are known to change their charge state depending on the applied gate voltage. The effects of such impurities lead to deviations from the simple capacitor model. Our evidence for such impurity states is the hysteresis observed in resistance vs. gate voltage measurements, consistent with previous studies that systematically investigated such charge traps and impurities.

F. Effect of initial doping of angle steering

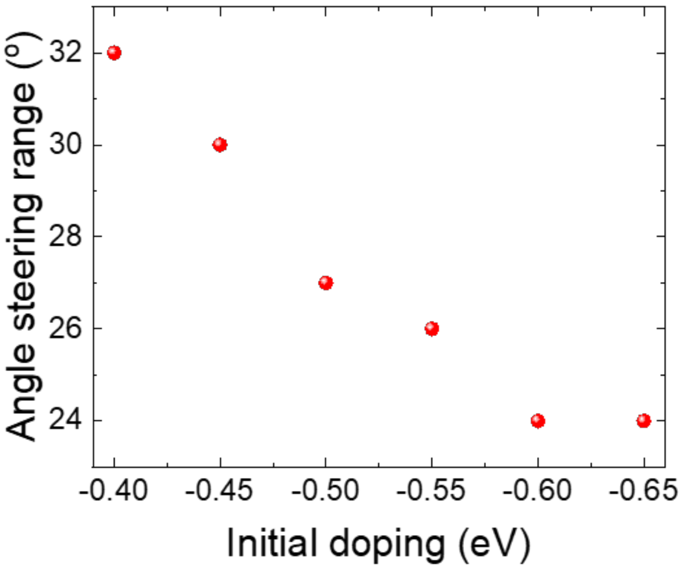

Fig. S19. Calculated angle steering range as a function of the initial doping level of graphene

The Fermi level-angular (Fig. 2(d)) and frequency-angular (Fig. S21) emissivity spectra indicate variations in both the steering angle range and modulation depth depending on the initial doping level of graphene. The correlation between the Fermi level and carrier density n_{carrier} follows the relationship $E_F \propto \sqrt{n_{\text{carrier}}}$, suggesting that higher Fermi levels undergo less change with the same increase in carrier density (proportional to V_G). Consequently, a higher initial doping level results in less alteration of the Fermi level for a given applied gate voltage. Figure S19 shows that the steering angle range for $E_F = -0.4\,\text{eV}$ at $V_G = 0$ is $6°$ higher than in the case of an initial doping of $-0.55\,\text{eV}$.

Supplementary Note 6. Potential angle steering capability

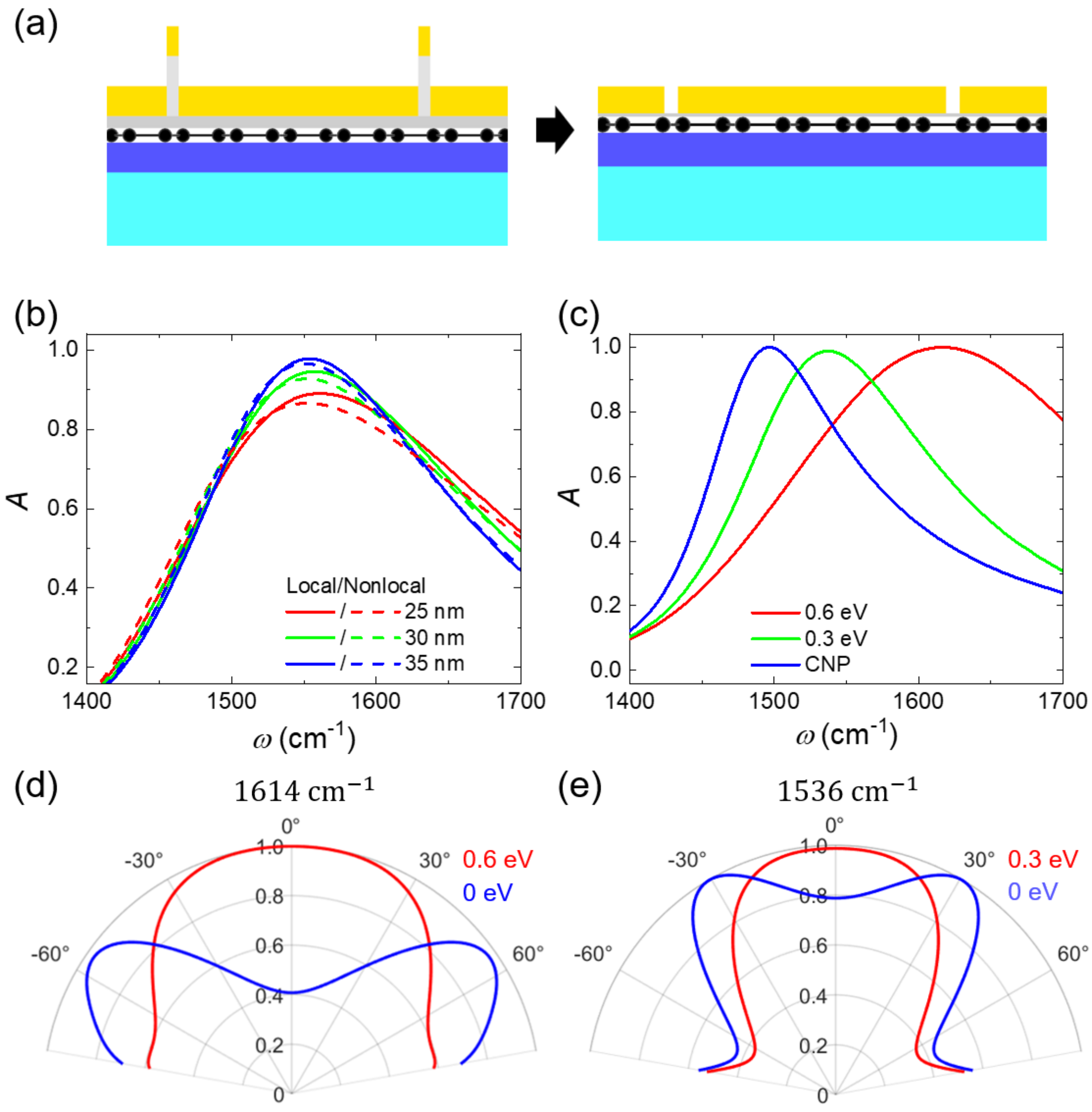

Fig. S20. Theoretical performance limit of the proposed configuration. (a) The schematic of geometry modification for optimization. (b) The total absorption of optimized structure ideal structure for different gap sizes and models with $E_F = 0.6\,\text{eV}$. (c) The total absorption of optimized for various Fermi levels of graphene. The angular absorption spectrum for (d) $0\,\text{eV}/0.6\,\text{eV}$ at $1614\,\text{cm}^{-1}$ and (e) $0\,\text{eV}/0.3\,\text{eV}$ at $1536\,\text{cm}^{-1}$.

In this study, We primarily focus on demonstrating dynamic control of directional emission angles, and the change of emission angles of the fabricated device was limited to $16°$. However, the proposed design scheme has the potential for a much larger emission angle change through the optimization of structural parameters and slight modifications to the configuration, as shown in Figure S20(a). The results of the structural parameter effect analysis indicate that the strength of interaction between graphene and the metal slit array strongly influences the emission angle θ. To maximize electromagnetic field

intensity at the surface of graphene, we propose ideal structure where HfO_2 layer, located on top of graphene, is reduced to $1\,\mathrm{nm}$. This configuration ensures the smallest distance between graphene and the metal slit array without the quantum tunneling effect. The gap and slit widths are optimized for the largest emission angle change.

Considering the significant degradation of performance due to the nonlocal effect for gaps less than $30\,\mathrm{nm}$, the minimum gap width is limited to $30\,\mathrm{nm}$. For Fermi level modulation at the $0\,\mathrm{eV}$ and $0.6\,\mathrm{eV}$, we obtain an emission angle change of approximately 60° for gap and slit widths of $30\,\mathrm{nm}$ and $740\,\mathrm{nm}$, respectively. Here, we focus on maximizing angle change. However, if the goal is to achieve emission steering with narrow beam, we can obtain a narrower beam by setting a high Fermi level at $0.3\,\mathrm{eV}$, as shown in Fig. S20(e). In this case, the maximum emission angle change is 40° due to reduced Fermi level modulation. Additionally, by employing other materials with smaller material loss than graphene, we anticipate the possibility of even narrower beam steering.

Supplementary Note 7. Modulation of angle-frequency spectra

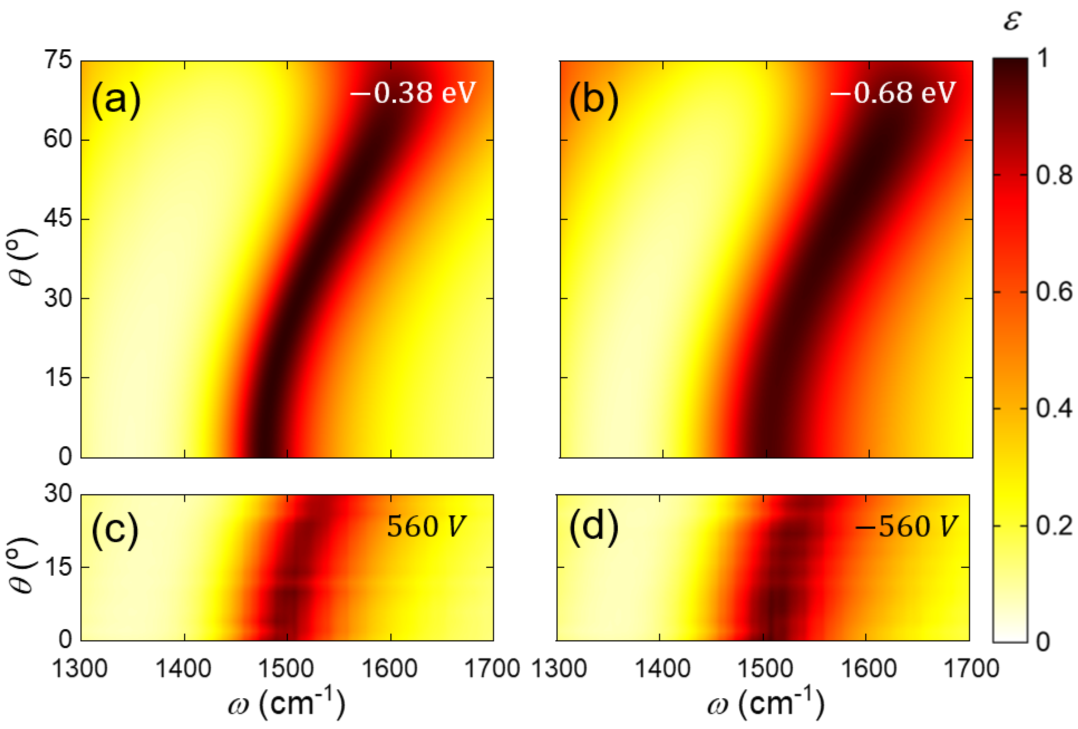

Fig. S21. Comparing calculated and measured emissivity map of the device. The calculated angle-frequency total emissivity spectra of the fabricated device for (a) $E_F = -0.38\,\text{eV}$ and (b) $-0.68\,\text{eV}$, respectively. Panels (c) and (d) show the measured angle-frequency emissivity spectra of the device for $V_G = 560$ V and -560 V, corresponding to each Fermi level.

To gain a deeper understanding of the device's operation, it is essential to analyze the emissivity behavior for the angle-frequency spectrum with fixed Fermi levels. Figures 21(a) and 21(b) depict the angle- and frequency-dependent emissivity spectra for $E_F = -0.38\,\text{eV}$ and $-0.68\,\text{eV}$ corresponding to $V_G = 560$ V and -560 V, respectively. The resonance frequency gradually shifts to a higher frequency with an increase in the incident angle, consistent with the previous result. The incident angle-dependent propagation phase accumulation is compensated by frequency-dependent phase change. It is noteworthy that the change in the Fermi level of graphene induces a simple translation of the resonance frequency curve. This constant frequency translation of the curve is inferred through nonresonant phase modulation of the graphene metasurface. The width of the resonance peak is broader for a higher Fermi level due to increased optical loss (see Supplementary Note 5). The experimentally measured angle- and frequency-dependent emissivity is well matched with the calculation results, as shown in Fig. S21(c) and (d).

References

[1] Min Seok Jang, Victor W. Brar, Michelle C. Sherrott, Josue J. Lopez, Laura Kim, Seyoon Kim, Mansoo Choi, and Harry A. Atwater. Tunable large resonant absorption in a midinfrared graphene salisbury screen. *Phys. Rev. B*, 90(5):165409, 2014.

[2] Seyoon Kim, Min Seok Jang, Victor W. Brar, Kelly W. Mauser, Laura Kim, and Harry A. Atwater. Electronically tunable perfect absorption in graphene. *Nano Lett.*, 18(2):971–979, 2018.

[3] Younes Ra'di, Constantin R. Simovski, and Sergei A. Tretyakov. Thin perfect absorbers for electromagnetic waves: Theory, design, and realizations. *Phys. Rev. Appl.*, 3:037001, Mar 2015.

[4] Farhan Rana. Graphene terahertz plasmon oscillators. *IEEE Transactions on Nanotechnology*, 7: 91, 2008.

[5] Haoliang Qian, Yuzhe Xiao, Dominic Lepage, Li Chen, and Zhaowei Liu. Quantum electrostatic model for optical properties of nanoscale gold films. *Nanophotonics*, 4:413, 2015.

[6] Kevin J. Savage, Matthew M. Hawkeye, Rubé'n Esteban, Andrei G. Borisov, Javier Aizpurua, and Jeremy J. Baumberg. Revealing the quantum regime in tunnelling plasmonics. *Nature*, 491:574, 2012.

[7] Wen Chen, Shunping Zhang, Meng Kang, Weikang Liu, Zhenwei Ou, Yang Li, Yexin Zhang, Zhiqiang Guan, and Hongxing Xu. Probing the ultimate limits of plasmonic enhancement. *Science*, 337:1072, 2012.

[8] A. D. Boardman. *Electromagnetic Surface Modes*. Wiley Chichester, 1982.

[9] Victor W. Brar, Michelle C. Sherrott, Min Seok Jang, Seyoon Kim, Laura Kim, Mansoo Choi, Luke A. Sweatlock, and Harry A. Atwater. Electronic modulation of infrared radiation in graphene plasmonic resonators. *Nat. Commun.*, 6(1):7032, 2015. ISSN 2041-1723.
